# Successful Case of Tracheotomy in Croup

**Published:** 1868

**Authors:** J. H. Wythe

**Affiliations:** Salem, Oregon


### Successful Case of Tracheotomy in Croup. By J. H. Wythe, M. D., Salem Oregon.

On November 28th, 1867, I was desired to hold a consultation with Dr. J. Boswell, respecting two children of Rev. N. Doane, residing in this place. One was a stout boy of twelve years of age, the other a girl of six years. Both were similarly affected, and in an advanced stage of croup. The disease was evidently inflammatory, and began first in ths tonsils, with no appearance of diphthetric exudation, and passed downwards, into the larnyx and trachea. In the little girl, the suffocative crisis seemed to have passed by the day before, and the disease was affecting the smaller bronchial tubes. In the boy, the larynx was most affected. In both, there was complete aphonia, ringing cough and stridulous breathing, with great restlessness and anxiety. They had been treated with ipecac, and tartar emetic, occasional laxative doses of calomel, sinapisms, etc. I suggested frequent doses of calomel, with a view to its effect on the plastic power of the blood, and referred to tracheotomy as a last resort.

The next evening, the 29th, I was sent for to operate on the boy. There was, however, considerable opposition to the procedure, and much valuable time was lost in considering objections. Dr. H. C. Carpenter was also sent for, and, assisted by him, the attending physician and my son, I opened the trachea. The boy at the time, was *in articulo mortis.* Deep coma had come on, and consciousness had been suspended for nearly half an hour. The pulse, thread-like and fluttering, could scarcely be felt under the figure. The features were pinched and hippocratic. The extremities were cold and blue, and the diaphragm was making irregular sposmodic efforts at respiration.

I made an incision two inches long through the skin, pushed aside the muscles and vessels with the handle of the knife, and on exposing the trachea, made an opening through it in the usual way, and placed in it a double silver canula. The effect of the operation was immediate. After one or two spasmodic inspirations, the pulse returned, and in a short time animal warmth and consciousness were fully restored.

The next morning, a strong solution of nitrate of silver—120 grains to the ounce—was passed, by a very flexible whalebone probang, into the trachea, both upwards and downwards, which brought away from the larynx several pieces of tough, white, fibrous membrane, and from the trachea a muco-purulent discharge.

A collapse of the lower lobe of the right lung remained for a time, and some difficulty was found during the treatment in keeping the silver tracheal tube free from inspissated mucus. A few drops of glycerin had the best effect for this purpose. Otherwise, the case progressed favorably. On the sixth day, the tube was removed and the wound dressed with adhesive strips. The patient has since remained in excellent health.

A few years ago, I performed the same operation on a child of Dr. Shoemaker, Lazaretto Physician at Philadelphia. She was suffering with diphtheria, and appeared almost at the last gasp. The opening of the trachea gave her great relief, but the disease continued, and exhausted the patient on the sixth day after the operation.

In another case, of an adult, suffocating from a laryngeal abscess, it was evidently the means of saving life.

These instances, in my own experience, lead me to regard tracheotomy with greater favor than authorities and statistics would seem to justify, and I should not hesitate to advise it in cases of imminent suffocation which had resisted a reasonable amount of medical treatment. It has, however, occurred to me that the introduction of a tube (as a catheter) into the trachea through the mouth, might be euccessfully resorted to in some cases. The chief objection to it would be its liability to increase the inflammation and œdema of the glottis and larynx.

